# Primary Chest Wall Ewing Sarcoma With Diaphragmatic Invasion in an Adult: En‐Bloc Resection and Polypropylene Mesh Reconstruction

**DOI:** 10.1002/ccr3.71824

**Published:** 2026-01-07

**Authors:** Mohammad Alaa Aldakak, Ahmad Al‐Bitar, Raneem Ahmad, Hamza Alhallaq, Kinda Assoud, Hussain Chaban

**Affiliations:** ^1^ Faculty of Medicine Damascus University Damascus Syrian Arab Republic; ^2^ Al‐Mouwasat University Hospital, Faculty of Medicine Damascus University Damascus Syrian Arab Republic

**Keywords:** chest wall, diaphragmatic invasion, en bloc resection, Ewing sarcoma, mesh reconstruction

## Abstract

Chest‐wall Ewing sarcoma (CWES) is uncommon in adults and often abuts vital thoracic structures, making R0 resection challenging despite gains with multimodal therapy. A 46‐year‐old Arab male presented with year‐long right‐sided chest pain, weight loss, and a firm mass over the lower right ribs. CXR showed a lateral pleural‐based opacity; computed tomography (CT) demonstrated a chest‐wall lesion involving right ribs 9–11 with diaphragmatic contact. CT‐guided core biopsy confirmed Ewing sarcoma. After 4 cycles of neoadjuvant multi‐agent chemotherapy, he underwent composite resection of ribs 9–11 with partial diaphragmatic excision and polypropylene (Prolene) mesh reconstruction stabilized by two wires. Pathology revealed a small round blue cell tumor with broad necrosis; margins were negative laterally, posteriorly, and at cartilaginous/diaphragmatic edges, but the anterior margin was positive (R1). IHC showed diffuse membranous CD99, nuclear FLI1, diffuse vimentin, and focal NSE; molecular confirmation of EWSR1 rearrangement was recommended. This adult CWES with diaphragmatic invasion illustrates the need for multimodal care: induction chemotherapy to address micrometastases and facilitate resection, followed by aggressive local surgery and prosthetic reconstruction. The R1 anterior margin justifies adjuvant systemic therapy with consideration of postoperative radiotherapy to optimize local control. Definitive diagnosis relies on integrated histology, immunophenotype, and molecular testing. Adult CWES may require combined rib and diaphragmatic resection with prosthetic repair. Margin‐negative surgery remains pivotal; when margins are positive, tailored adjuvant therapy is essential to mitigate local and micrometastatic risks.

## Key Clinical Message

Adult CWES/PNET may necessitate combined rib and diaphragmatic resection with prosthetic reconstruction; even after neoadjuvant therapy, an R1 margin should trigger adjuvant chemotherapy and consideration of postoperative radiotherapy to optimize local control.

## 1 Introduction

Ewing's sarcoma family tumors (ESFT) is a highly aggressive malignant small round blue cell tumor and the second most common primary bone cancer affecting children, adolescents, and young adults [1]. While it can arise in nearly any bone or soft tissue, primary manifestation in the chest wall (CWES) is relatively rare, posing significant diagnostic and therapeutic challenges due to the complex anatomy and proximity to vital thoracic organs [2, 3]. The clinical presentation can be varied, often mimicking more benign conditions, which can, unfortunately, lead to delays in diagnosis [1]. The management of CWES has undergone significant evolution over the past several decades, with survival rates exhibiting marked improvement [4, 5, 6]. The current standard of care is a comprehensive, multimodal approach that integrates systemic chemotherapy, surgery, and radiotherapy [5]. Surgical intervention with the goal of complete en bloc resection with negative microscopic margins (R0 resection) is a cornerstone of curative‐intent therapy and one of the most important factors for long‐term survival [5, 6, 7, 8]. However, achieving this in the chest wall often requires extensive resections that necessitate complex reconstructions to maintain thoracic stability and respiratory function [3, 9]. Despite these advances, the prognosis for patients with CWES can be guarded. The presence of metastatic disease at diagnosis remains the most significant adverse prognostic factor [6]. Here, we present the case of a 46‐year‐old Arab male diagnosed with Ewing's sarcoma of the chest wall.

## 2 Case Presentation

### 2.1 Case History/Examination

**FIGURE 1:**
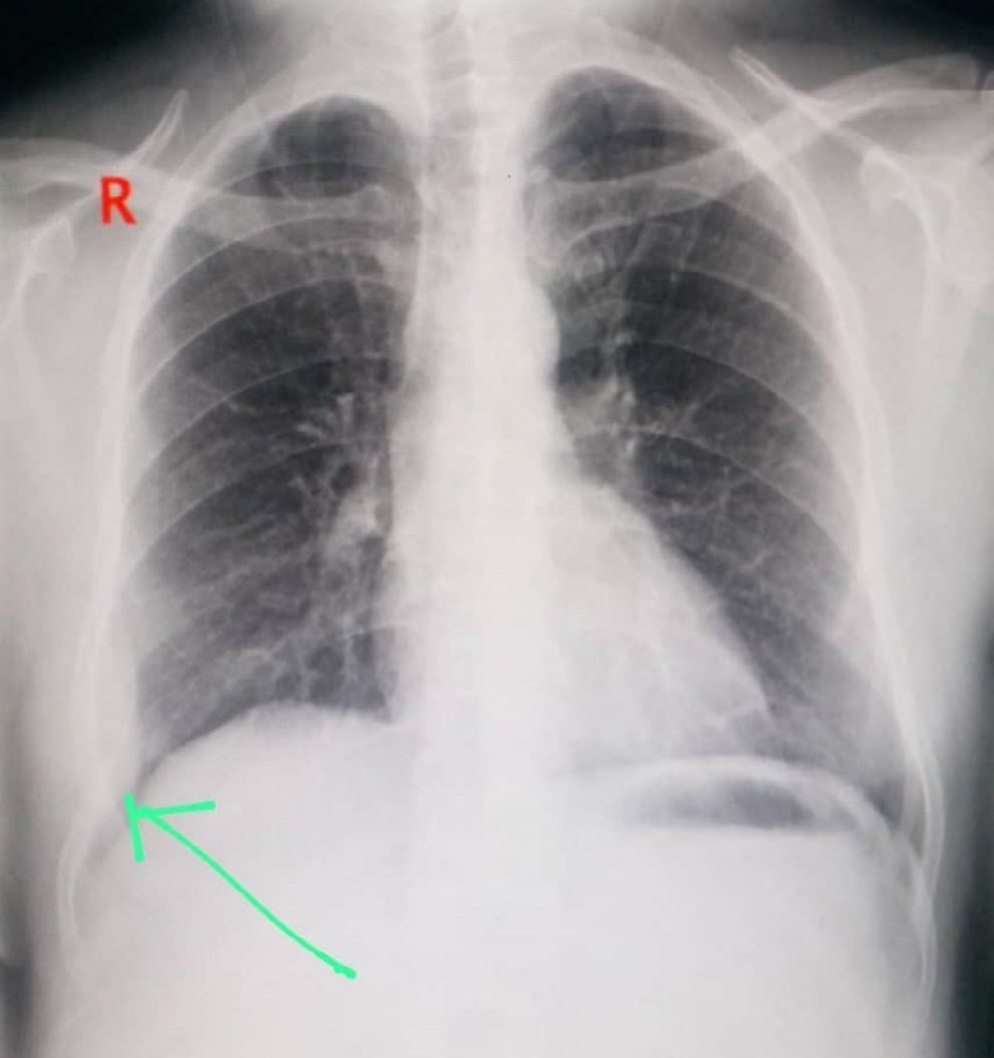
Posteroanterior chest radiograph. A pleural‐based opacity projects over the right lower hemithorax along the lateral chest wall (green arrow), suspicious for a rib‐origin lesion. Cardiomediastinal contours are preserved; no focal parenchymal consolidation is seen.

**FIGURE 2:**
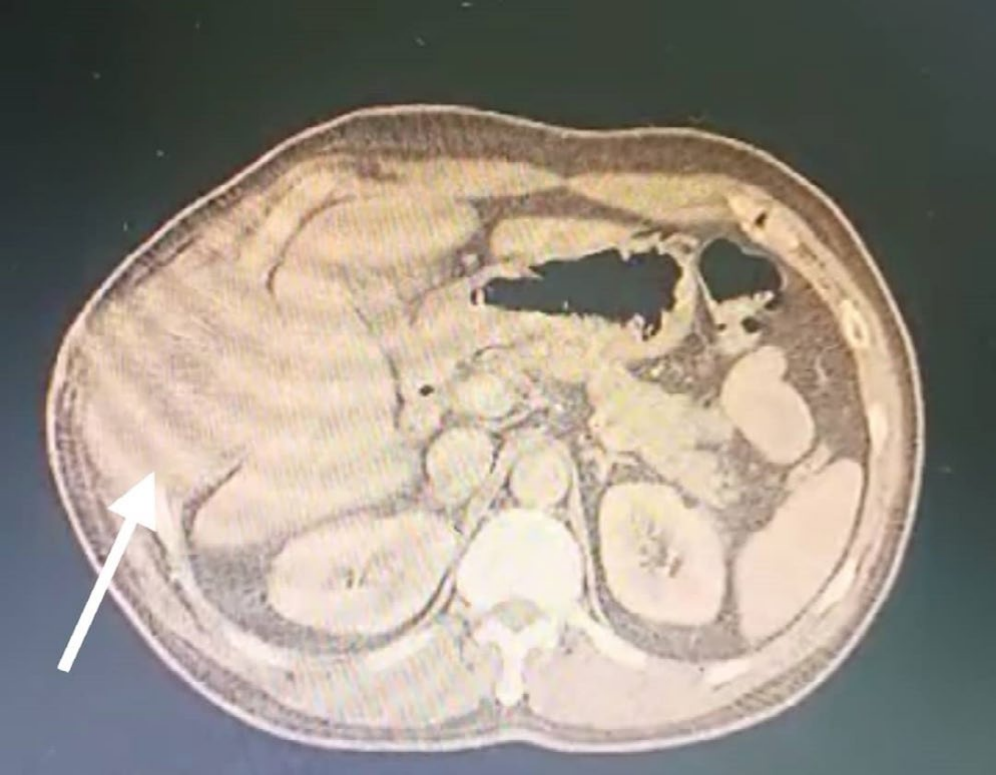
Axial CT of the lower thorax. Right posterolateral chest wall mass arising from the ribs (white arrow), with cortical irregularity/erosion and an associated soft‐tissue component. The lesion involves the right 9th–11th ribs in continuity.

**FIGURE 3:**
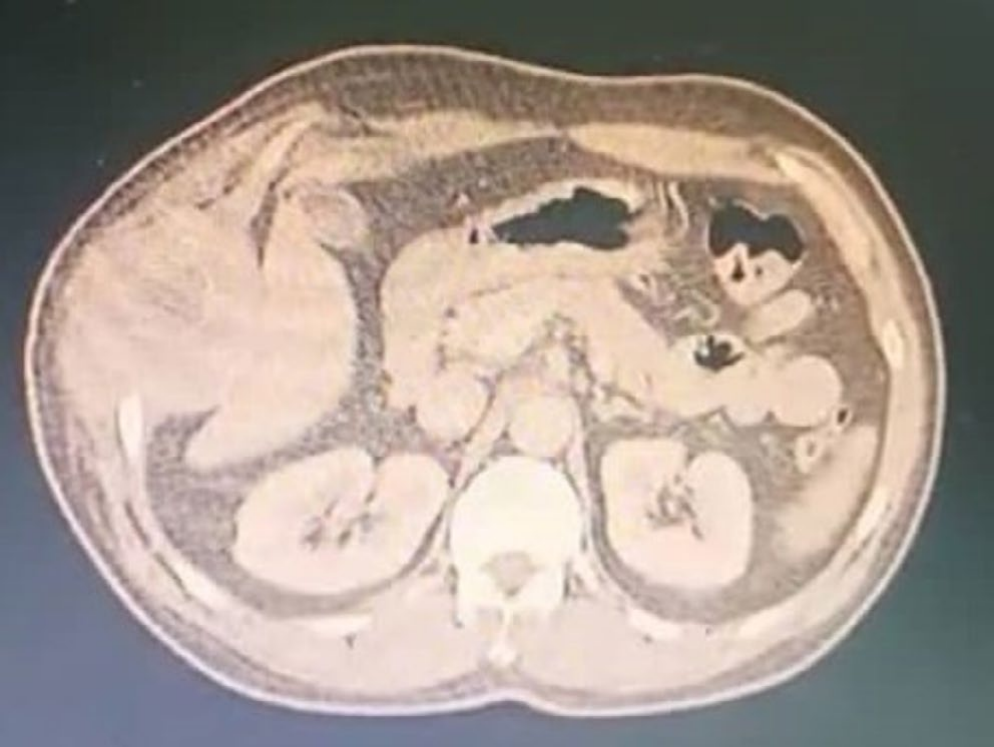
Axial CT at a slightly different level. The chest wall mass remains in broad contact with the right hemidiaphragm, with effacement of the intervening fat plane—features concerning diaphragmatic involvement. No pleural effusion is evident on these cuts.

We present a 46‐year‐old Arab married male laborer with a 40‐pack‐year smoking history who does not consume alcohol. He presented with persistent right‐sided chest pain unresponsive to analgesics. His past medical history is notable for hypertension and Huntington's disease; surgically, he underwent cardiac catheterization with stent placement 1 year ago. He takes Propranolol and Carbamazepine, has no known allergies, and no contributory family history. Review of systems was positive for involuntary choreiform movements related to Huntington's disease, otherwise unremarkable. The present illness began about a year earlier with stabbing pain involving the right chest wall and right hypochondrium, extending to the right flank, accompanied by loss of appetite and approximately 6 kg weight loss over 1 month. Subsequently, he noticed swelling over the lower right ribs without localized heat or redness. On examination, vital signs were stable (BP 130/70 mmHg, HR 75 bpm, temperature 37°C, SpO_2_ 95% on room air). There was a visible swelling over the lower right ribs; palpation revealed a hard, non‐tender mass fixed to deep structures, and chorea was observed. The remainder of the general, cardiovascular, abdominal, and musculoskeletal examinations was within normal limits. Pre‐admission laboratory tests were reported as normal. A chest radiograph revealed a mass within a rib (Figure 1). Initial cardiac workup including ECG and echocardiogram was normal. Chest computed tomography (CT) demonstrated a chest wall lesion involving the 9th, 10th, and 11th right ribs (Figures 2 and 3). A CT‐guided biopsy confirmed CWES. He was referred for chemotherapy and completed 4 cycles, with the last cycle 1 month prior to admission to our hospital a month ago.

### 2.2 Differential Diagnosis

The presentation of a hard, fixed chest wall mass associated with weight loss and multi‐rib involvement on CT prompts consideration of primary malignant chest wall tumors such as CWES/PNET, chondrosarcoma, and osteosarcoma; metastatic disease to the ribs from primaries such as lung, breast, renal, thyroid, or prostate; hematologic malignancies including plasmacytoma/multiple myeloma and lymphoma; as well as benign or infectious entities like fibrous dysplasia, osteochondroma, enchondroma, and osteomyelitis. In this case, the clinical course, imaging findings, and tissue diagnosis converge on CWES as the definitive etiology.

## 3 Conclusion and Results (Outcome and Follow‐Up)

**FIGURE 4:**
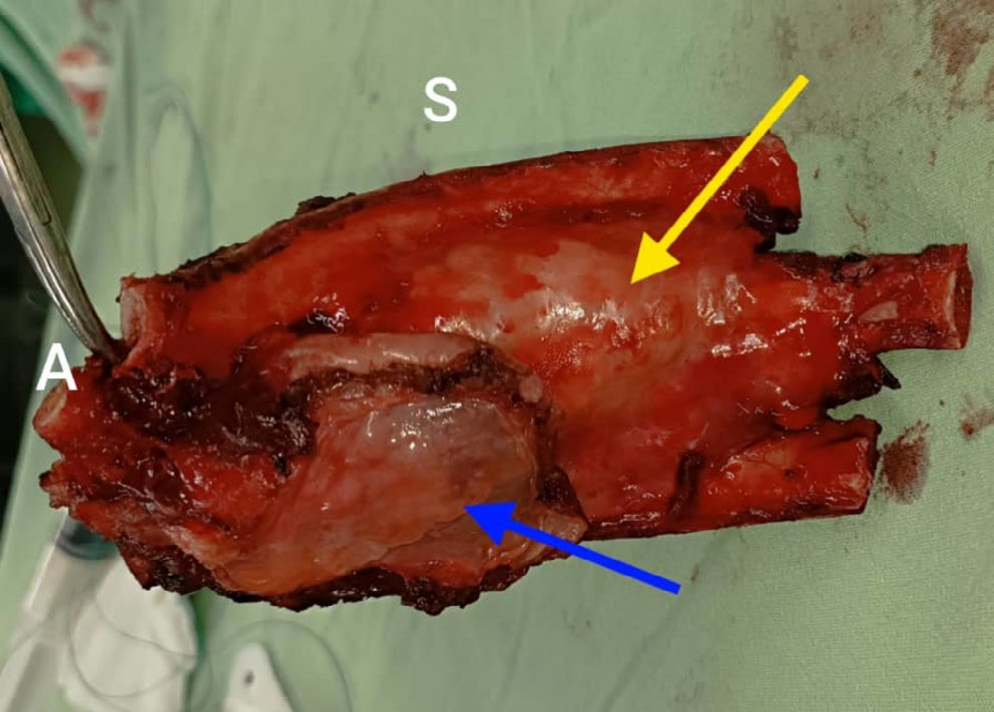
Intraoperative chest wall reconstruction. After composite resection of right ribs 9–11 with a diaphragmatic cuff, a polypropylene (Prolene) mesh is inserted to bridge the defect. Two stainless‐steel stabilizing wires traverse the mesh and are anchored to the residual rib ends (white arrows). Orientation: S, superior; A, anterior.

**FIGURE 5:**
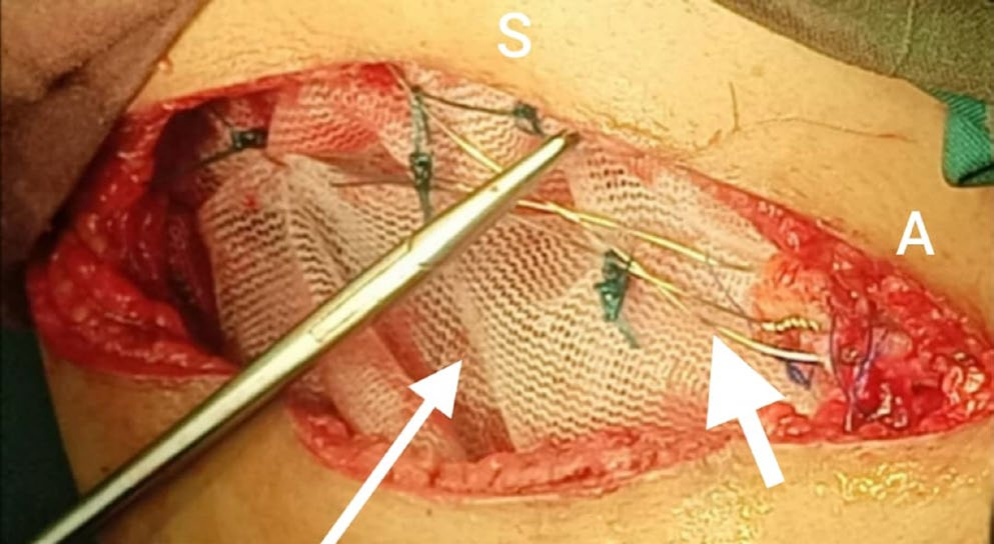
En‐bloc chest wall specimen. Gross specimen including segments of right ribs 9–11 with attached soft tissues. A tan‐white mass arising from the rib with an extraosseous component is evident (blue arrow), with focal cortical erosion on the external rib surface (yellow arrow).

**FIGURE 6:**
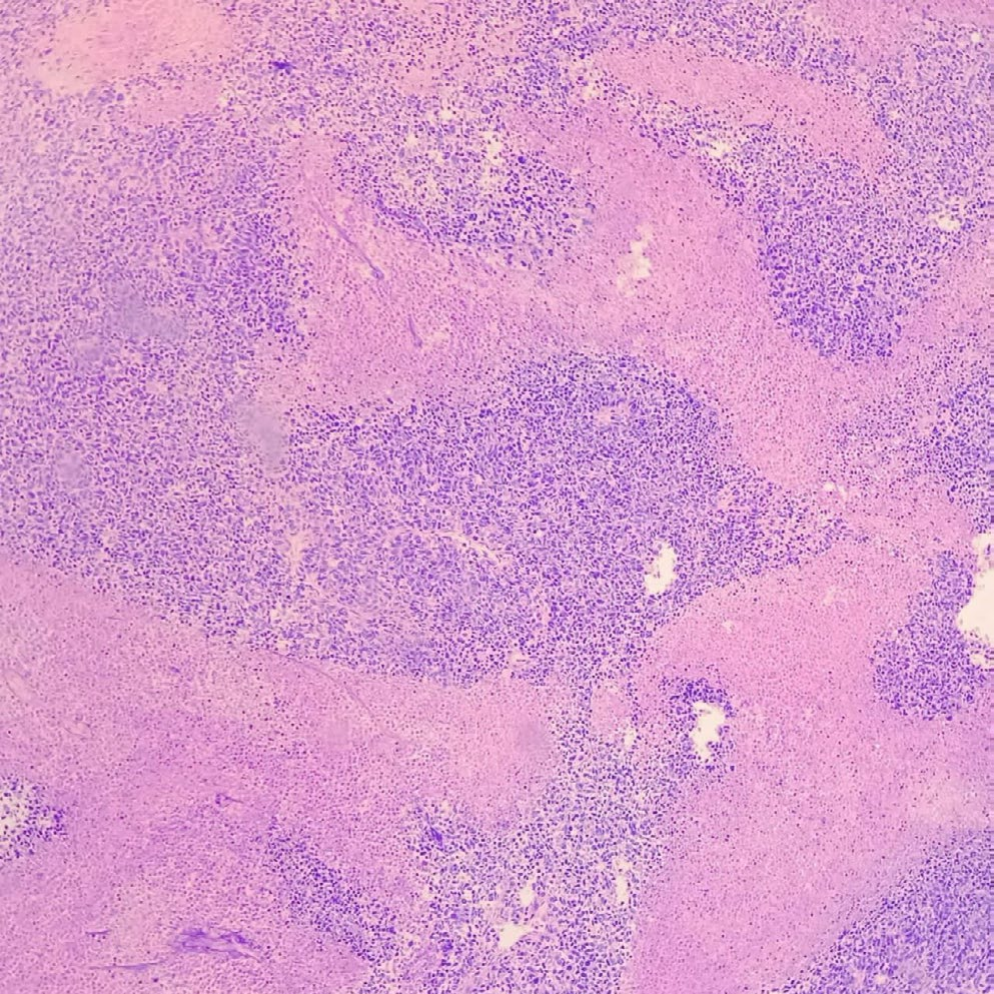
H&E, ×100. Sheets of monotonous small round blue cells with extensive geographic necrosis.

**FIGURE 7:**
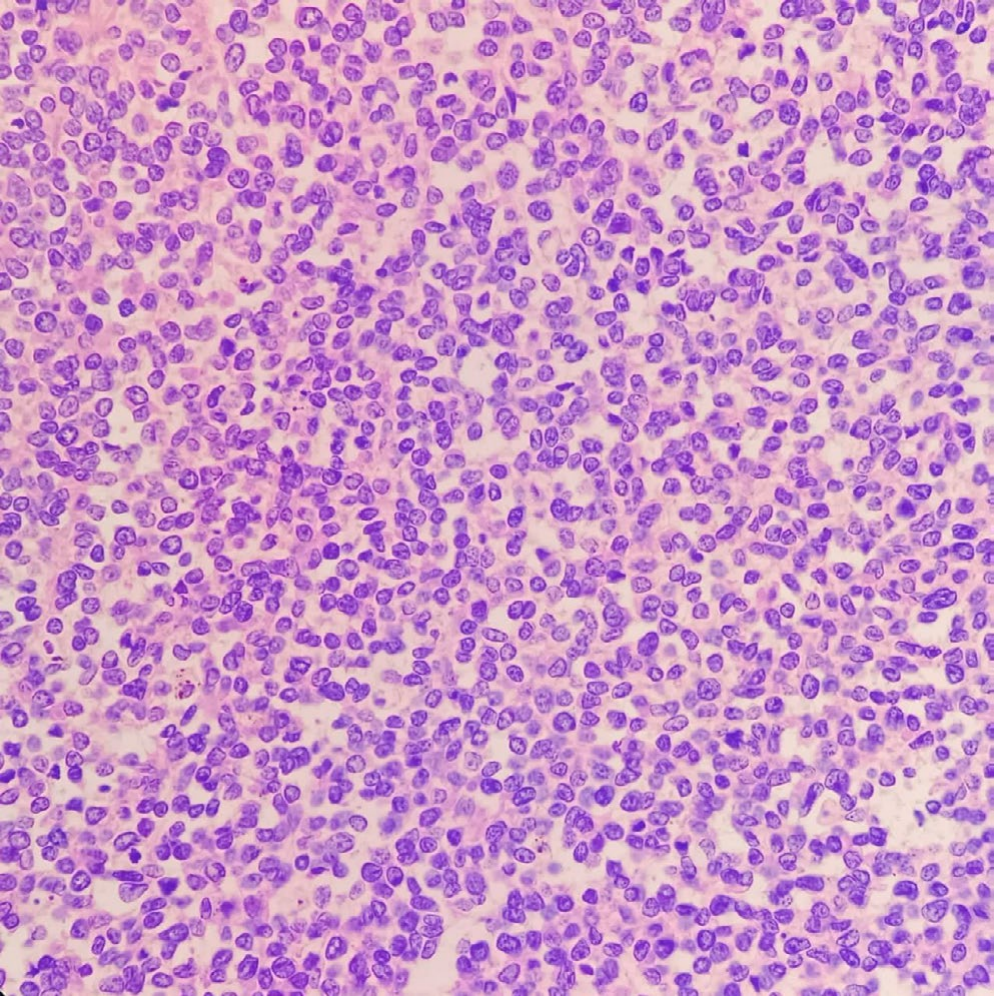
H&E, ×200. Tumor cells with scant cytoplasm, round to oval open chromatin nuclei, inconspicuous nucleoli, and brisk mitotic activity.

**FIGURE 8:**
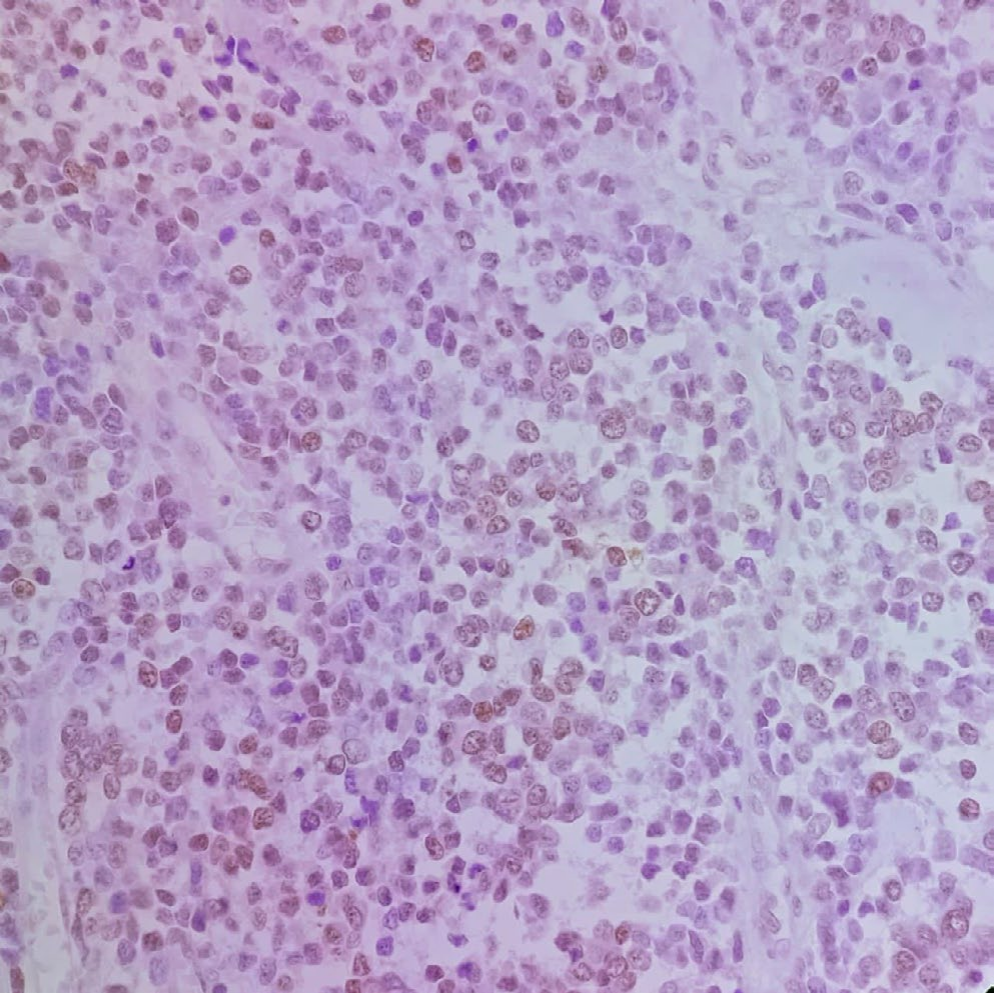
FLI1 immunohistochemistry, ×200. Diffuse nuclear positivity in tumor cells.

**FIGURE 9:**
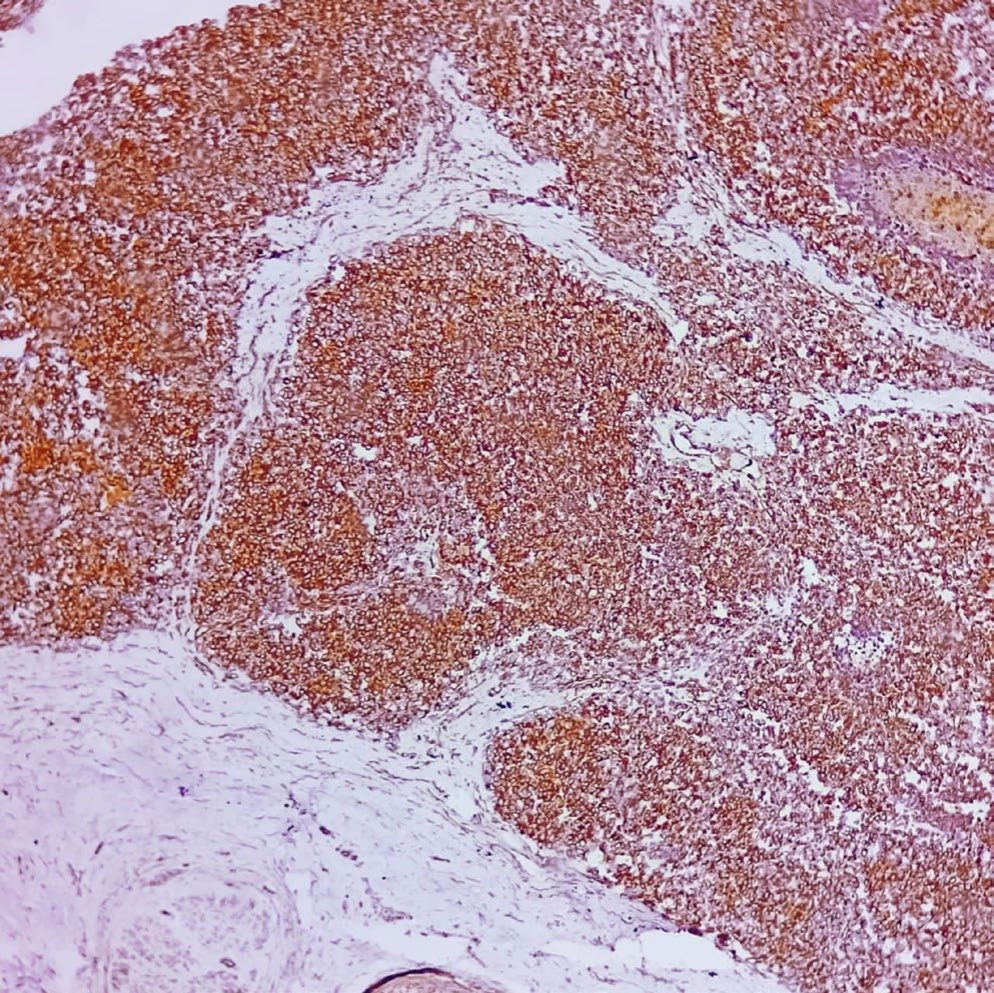
Vimentin immunohistochemistry, ×200. Diffuse cytoplasmic positivity within tumor cells.

**FIGURE 10:**
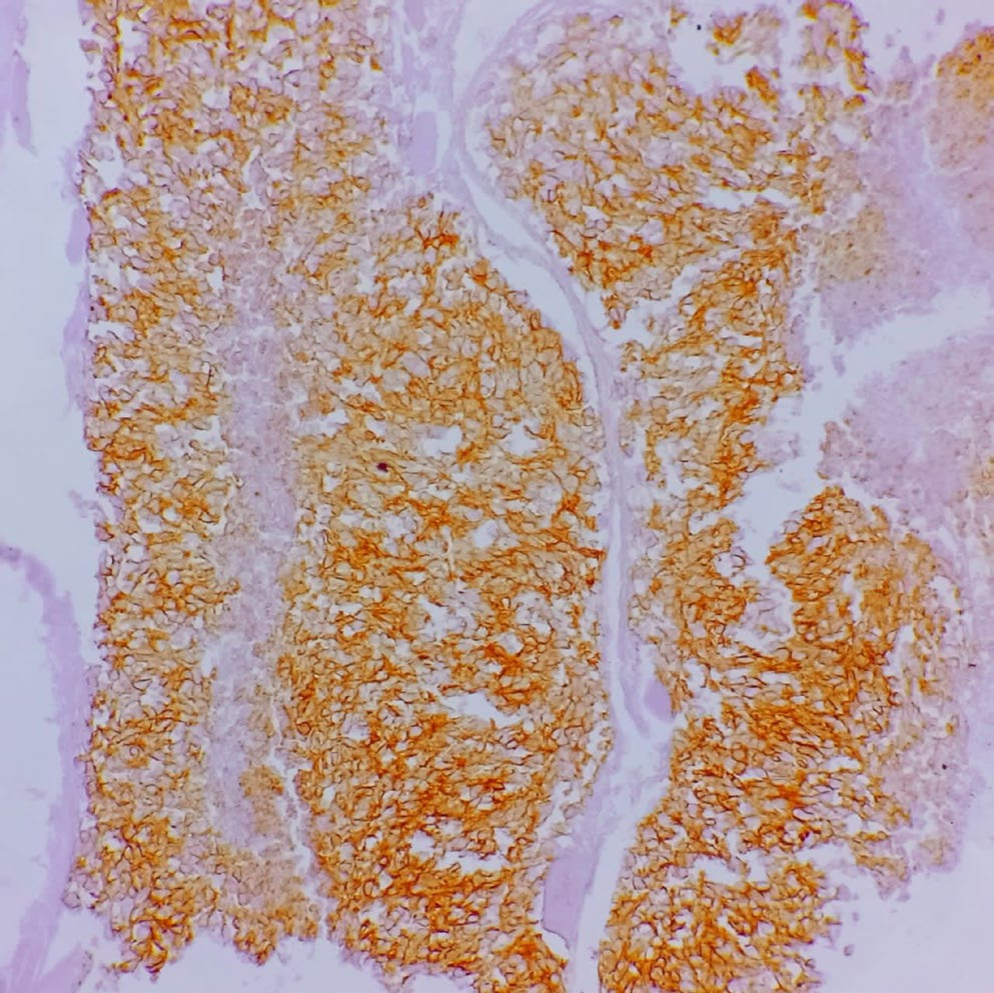
CD99 immunohistochemistry, ×200. Strong, predominantly membranous positivity.

**FIGURE 11:**
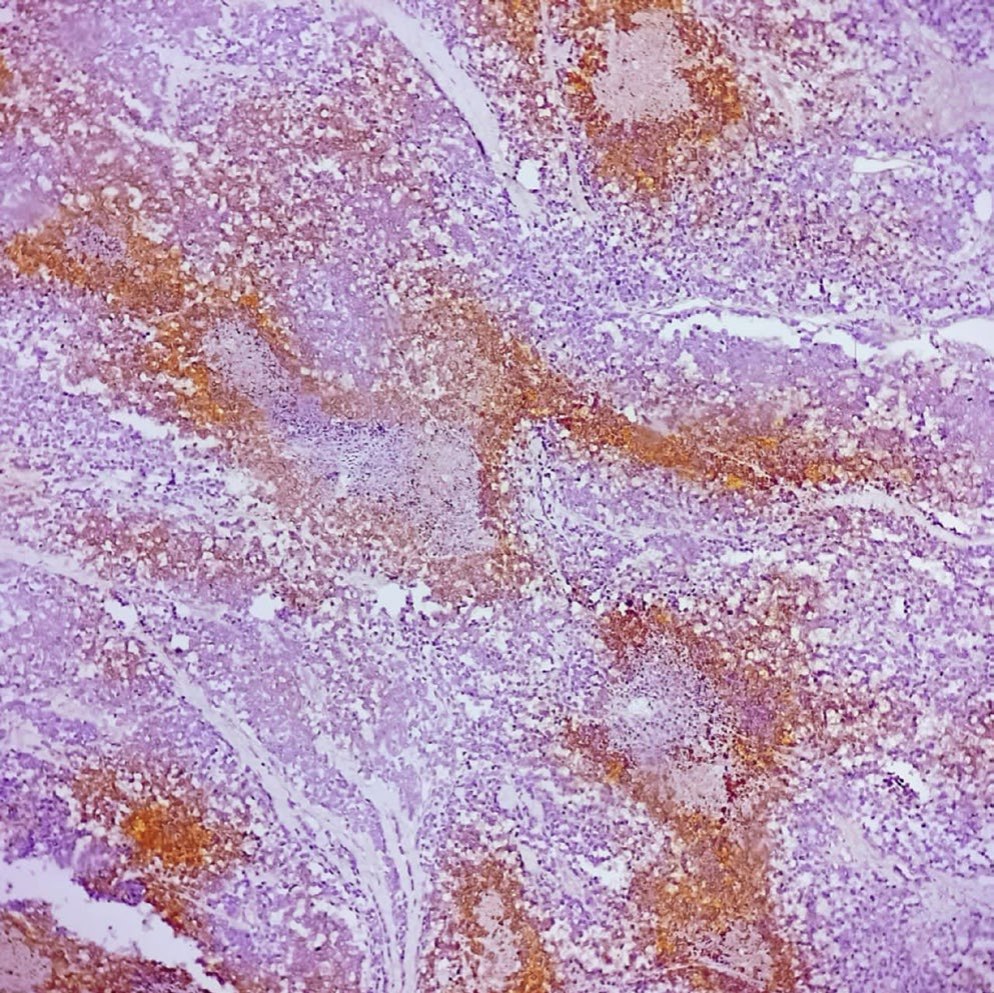
NSE immunohistochemistry, ×200. Focal cytoplasmic positivity in tumor cells.

The patient underwent surgical resection consisting of wide local excision of the chest wall mass with removal of the involved segments of the 9th, 10th, and 11th right ribs with 4 cm margins, along with resection of the diaphragmatic portion involved by tumor. Reconstruction of the resultant defect was achieved using a Prolene mesh with stabilization by two metal wires (Figure 4). Final pathology described a thoracic wall mass measuring 17 × 8 × 5.5 cm, including three ribs and part of the diaphragm (Figure 5). Histology showed an infiltrative small round blue cell neoplasm arranged in solid sheets and nests with broad geographic necrosis. Resection margins were negative at the diaphragmatic, lateral, cartilaginous, and posterior aspects, while the anterior margin was involved by tumor. Immunohistochemistry demonstrated diffuse strong membranous CD99 expression, nuclear FLI1 positivity, diffuse vimentin reactivity, and focal NSE positivity. In the morphologic and clinical context, this immunophenotype supports a diagnosis of CWES/PNET, with molecular confirmation of EWSR1 rearrangement (Figures 6, 7, 8, 9, 10, 11). Postoperative oncologic follow‐up is indicated given the positive anterior margin and the preoperative course of four chemotherapy cycles.

## 4 Discussion

Ewing sarcoma family tumors (ESFT) comprise malignant small round blue cell neoplasms with variable neuroectodermal differentiation, encompassing osseous EW, extra‐skeletal EW, peripheral primitive neuroectodermal tumor, and the chest‐wall (Askin) variant [10]. Since J. Ewing's original description in 1921 [11], ESFT were recognized primarily in adolescents, with a peak incidence at 13–16 years and a male predominance of roughly 2:1 [12]. Chest‐wall involvement typically arises from the ribs—affected in about 10% of all ESFT cases [13]—but primary malignant chest‐wall tumors themselves are uncommon, representing < 1% of primary tumors overall [14]. Beyond the chest wall and skeletal sites, Ewing sarcoma family tumors (ESFT) have been reported in a wide spectrum of anatomic locations, including genitourinary organs (kidney, adrenal gland, urinary bladder, ureter, prostate, penis, seminal vesicle, testis), gastrointestinal and hepatobiliary sites (small bowel, rectum, liver, gallbladder, pancreas), and head, neck, and thoracic structures (maxillary sinus, trachea, lung, parotid gland), as well as gynecologic and breast sites (vulva, vagina, ovary, uterine cervix, uterus, and breast) [15] Clinically, ESFT is highly aggressive, characterized by a propensity for local recurrence and early metastatic spread, and is therefore best approached as a systemic disease at presentation [16]. The earliest manifestation is typically progressively worsening pain, which may be accompanied by cough, fever, malaise, anemia, and an elevated erythrocyte sedimentation rate [12]. Compared with the adolescent predominance of ESFT, our case occurred in an adult but arose from the ribs—consistent with the ~10% chest‐wall involvement. It demonstrated aggressive local behavior (diaphragmatic invasion and an anterior positive margin), requiring composite chest‐wall/diaphragm resection and mesh reconstruction, reflecting the disease's systemic biology.

Given the aggressiveness of bone tumors, early recognition is critical, yet delays are common due to late presentation, low clinical suspicion, and nonspecific symptoms that mimic benign musculoskeletal injuries. Plain radiography remains the initial imaging modality of choice [17]. A properly planned biopsy is essential to establish the diagnosis, determine histologic grade, and direct therapy. For symptomatic bone pain—especially in children—magnetic resonance imaging (MRI) is the principal diagnostic study [18]; when MRI is indeterminate, projection radiography or CT should be undertaken [19].

Confirming primary CWES/PNET requires integrated histomorphology, immunophenotyping, and molecular testing. On H&E, the tumor shows solid sheets of small round blue cells with a high nuclear‐to‐cytoplasmic ratio and scant eosinophilic cytoplasm, features that overlap with embryonal rhabdomyosarcoma, neuroblastoma, lymphoma, and, particularly in adults with thoracic masses, small cell lung carcinoma. CD99 (MIC2) is a valuable screening marker and is often strongly positive in reported mediastinal cases, but it is neither fully specific (e.g., it can be positive in synovial sarcoma, lymphoblastic lymphoma, ependymoma, and other small round blue cell tumors) nor fully sensitive (rare Ewing sarcoma cases may show only weak or even negative CD99 staining). Therefore, confirmatory genetic analysis—most commonly demonstrating an EWSR1 rearrangement—is indispensable for a definitive diagnosis [3]. Our case aligns with this framework: classic SRBCT morphology on H&E with diffuse membranous CD99, nuclear FLI1, diffuse vimentin, and focal NSE—supportive yet non‐specific. Accordingly, EWSR1 rearrangement testing remains required for definitive confirmation (recommended/pending).

Optimal management of CWES/PNET is multimodal, combining systemic chemotherapy with definitive local control. Induction (neoadjuvant) chemotherapy—most commonly VDC/IE (vincristine, doxorubicin, cyclophosphamide alternating with ifosfamide and etoposide), with variants incorporating cyclophosphamide, doxorubicin, vincristine, etoposide, ifosfamide, and actinomycin‐D—targets micrometastatic disease, downstages the primary tumor, and permits assessment of histologic response, a key prognostic marker [1, 6, 20]. Local disease is managed with surgery, radiotherapy, or both; when resection is feasible, the goal is complete excision with negative (R0) margins, accepting wider or more complex resections when critical anatomy precludes safe clearance and complementing with adjuvant therapy as needed [21, 22]. Intensified multi‐agent regimens and meticulous local control have significantly improved survival in localized disease. Prognosis is influenced by tumor site, patient age, tumor volume, presence of metastases, and response to induction therapy [3, 23]. In our case, definitive local control was pursued with composite chest‐wall and diaphragmatic resection plus polypropylene‐mesh reconstruction, aligning with the principle of aggressive local therapy in CWES/PNET. However, upfront surgery (rather than neoadjuvant VDC/IE) yielded an anterior R1 margin; consequently, adjuvant multi‐agent chemotherapy with consideration of postoperative radiotherapy to the chest‐wall bed is warranted to optimize local control and mitigate micrometastatic risk—particularly given adult age, rib‐origin disease, and substantial tumor extent.

## 5 Conclusion

Adult CWES/PNET can present with extensive rib involvement and diaphragmatic invasion, complicating the attainment of negative margins. In this case, multi‐agent neoadjuvant chemotherapy was followed by composite chest‐wall and partial diaphragmatic resection with polypropylene (Prolene) mesh reconstruction; despite this, an anterior R1 margin necessitates adjuvant systemic therapy with consideration of postoperative radiotherapy. The case underscores the primacy of margin‐negative resection when feasible, the value of standardized immunohistochemistry with molecular confirmation, and the need for coordinated multidisciplinary care to optimize local control while preserving thoracic function.

## Author Contributions

**Mohammad Alaa Aldakak:** writing – original draft, writing – review and editing. **Ahmad Al‐Bitar:** writing – original draft. **Raneem Ahmad:** writing – original draft, writing – review and editing. **Hamza Alhallaq:** visualization, writing – review and editing. **Kinda Assoud:** data curation, investigation. **Hussain Chaban:** supervision, writing – review and editing.

## Funding

The authors have nothing to report.

## Ethics Statement

Institutional Review Board (IRB) approval is not required for de‐identified single case reports or case histories, in accordance with institutional policies.

## Consent

Written informed consent was obtained from the patient for publication and any accompanying images. A copy of the written consent is available for review by the Editor‐in‐Chief of this journal on request.

## Conflicts of Interest

The authors declare no conflicts of interest.

## Data Availability

Data available on request from the authors.

## References

[1] A. Al‐Bitar and M. Sandouk , “Maxillary Ewing Sarcoma in a Teenager: A Case Report,” Case Reports in Oncology 18, no. 1 (2025): 515–522, 10.1159/000545490. 40302989 PMC12040304

[2] I. Sakharuk , T. McKinley , G. Moore , and D. Miller , “Ewing Sarcoma–Chest Wall Reconstruction Following Resection of Rare Primary Chest Wall Tumor,” American Surgeon 90, no. 7 (2024): 1942–1944, 10.1177/00031348241241635. 38532255

[3] C. Su , X. Zhu , and J. Zhang , “Primary Mediastinal Ewing's Sarcoma Presenting With Sudden and Severe Chest Pain: A Case Report,” Frontiers in Oncology 13 (2024): 1290603, 10.3389/fonc.2023.1290603. 38282670 PMC10811232

[4] S. K. Qupp , I. M. Halayqa , E. J. Shawahna , et al., “Primary Ewing's Sarcoma of the Sternum in an Adult Male: A Rare Case Report,” Journal of Investigative Medicine High Impact Case Reports 12 (2024): 23247096241286358, 10.1177/23247096241286358. 39369316 PMC11457185

[5] B. Provost , G. Missenard , C. Pricopi , et al., “Ewing Sarcoma of the Chest Wall: Prognostic Factors of Multimodal Therapy Including en Bloc Resection,” Annals of Thoracic Surgery 106, no. 1 (2018): 207–213, 10.1016/j.athoracsur.2018.02.031. 29551629

[6] A. J. Jacobs , J. Fishbein , C. F. Levy , and R. D. Glick , “Chest Wall Ewing Sarcoma: A Population‐Based Analysis,” Journal of Surgical Research 204, no. 2 (2016): 475–480, 10.1016/j.jss.2016.05.033. 27565085

[7] J. W. Denbo , W. Shannon Orr , Y. Wu , et al., “Timing of Surgery and the Role of Adjuvant Radiotherapy in Ewing Sarcoma of the Chest Wall: A Single‐Institution Experience,” Annals of Surgical Oncology 19, no. 12 (2012): 3809–3815, 10.1245/s10434-012-2449-5. 22752372 PMC3529468

[8] R. C. Shamberger , M. P. Laquaglia , M. D. Krailo , et al., “Ewing Sarcoma of the Rib: Results of an Intergroup Study With Analysis of Outcome by Timing of Resection,” Journal of Thoracic and Cardiovascular Surgery 119, no. 6 (2000): 1154–1161, 10.1067/mtc.2000.106330. 10838532

[9] I. Simal , M. A. García‐Casillas , J. A. Cerdá , et al., “Three‐Dimensional Custom‐Made Titanium Ribs for Reconstruction of a Large Chest Wall Defect,” European Journal of Pediatric Surgery Reports 4, no. 1 (2016): 26–30, 10.1055/s-0036-1593738. 28018805 PMC5177554

[10] D. Mathew , D. N. Prince , and N. Mahomed , “Extra‐Skeletal Ewing Sarcoma of the Chest Wall in a Child,” South African Journal of Radiology 23, no. 1 (2019): 1733, 10.4102/sajr.v23i1.1733. 31754538 PMC6837769

[11] C. Karatziou , X. Pitta , T. Stergiouda , V. Karadimou , and G. Termentzis , “A Case of Extraskeletal Ewing Sarcoma Originating From the Visceral Pleura,” Hippokratia 15, no. 4 (2011): 363–365. 24391423 PMC3876857

[12] O. Salimbene , D. Viggiano , F. Muratori , et al., “Primary Chest Wall Ewing Sarcoma: Treatment and Long‐Term Results,” Life (Basel) 14, no. 6 (2024): 766, 10.3390/life14060766. 38929749 PMC11204814

[13] V. M. Thakre , V. Athawale , and T. Fating , “Adherence and Satisfaction With Intensive Physiotherapy Treatment During Ongoing Chemotherapy Sessions in Patients With Chest Wall Ewing Sarcoma,” Cureus 16, no. 1 (2024): e52289, 10.7759/cureus.52289. 38357048 PMC10865281

[14] J. A. Roth , J. C. Ruckdeschel , and T. H. Weisenburger , Thoracic Oncology (WB Saunders, 1995).

[15] N. Koufopoulos , S. Kokkali , D. Manatakis , et al., “Primary Peripheral Neuroectodermal Tumor (PNET) of the Adrenal Gland: A Rare Entity,” Journal of BUON 24, no. 2 (2019): 770–778. 31128035

[16] G. Ahmed , M. Zamzam , M. S. Zaghloul , et al., “Outcome of Resectable Pediatric Ewing Sarcoma of the Ribs,” Journal of the Egyptian National Cancer Institute 29, no. 2 (2017): 99–104, 10.1016/j.jnci.2017.03.002. 28462848

[17] J. L. Ferguson and S. P. Turner , “Bone Cancer: Diagnosis and Treatment Principles,” American Family Physician 98, no. 4 (2018): 205–213. 30215968

[18] D. Daniel, Jr. , E. Ullah , S. Wahab , and V. Kumar, Jr. , “Relevance of MRI in Prediction of Malignancy of Musculoskeletal System—A Prospective Evaluation,” BMC Musculoskeletal Disorders 10 (2009): 125, 10.1186/1471-2474-10-125. 19811663 PMC2766372

[19] G. Ulaner , S. Hwang , R. A. Lefkowitz , J. Landa , and D. M. Panicek , “Musculoskeletal Tumors and Tumor‐Like Conditions: Common and Avoidable Pitfalls at Imaging in Patients With Known or Suspected Cancer. Part A: Benign Conditions That May Mimic Malignancy,” International Orthopaedics 37, no. 5 (2013): 871–876, 10.1007/s00264-013-1823-7. 23436133 PMC3631500

[20] J. Haeusler , A. Ranft , T. Boelling , et al., “The Value of Local Treatment in Patients With Primary, Disseminated, Multifocal Ewing Sarcoma (PDMES),” Cancer 116, no. 2 (2010): 443–450, 10.1002/cncr.24740. 19924786

[21] T. Ozaki , “Diagnosis and Treatment of Ewing Sarcoma of the Bone: A Review Article,” Journal of Orthopaedic Science 20, no. 2 (2015): 250–263, 10.1007/s00776-014-0687-z. 25691401 PMC4366541

[22] M. A. Aldakak , A. Yassin , W. Hasan , and A. Al‐Bitar , “Primary Monophasic Synovial Sarcoma of the Plantar Foot With Pulmonary Metastases in a Young Adult: A Rare Case Report,” Case Reports in Oncology 18 (2025): 1361–1366, 10.1159/000548598. 41323059 PMC12659198

[23] M. A. Aldakak , A. Dayoub , A. Al‐Bitar , and A. Solaiman , “Giant Spindle Cell Lipoma of the Left Inguinal Region: A Rare Case With Diagnostic Challenges on MRI,” Radiology Case Reports 20 (2025): 4262–4265, 10.1016/j.radcr.2025.05.034. 40547940 PMC12182281

